# Sapitinib: reactive intermediates and bioactivation pathways characterized by LC-MS/MS†

**DOI:** 10.1039/c9ra03926k

**Published:** 2019-10-16

**Authors:** Mohamed W. Attwa, Adnan A. Kadi

**Affiliations:** Department of Pharmaceutical Chemistry, College of Pharmacy, King Saud University P. O. Box 2457 Riyadh 11451 Saudi Arabia mzeidan@ksu.edu.sa +966 1146 76 220 +966 1146 70237; Students' University Hospital, Mansoura University Mansoura 35516 Egypt

## Abstract

Sapitinib (AZD8931, SAP) is an epidermal growth factor receptor (EGFR) family (pan-erbB) tyrosine kinase inhibitor. In multiple tumor cell lines, SAP has been shown to be a much more potent inhibitor of EGF-driven cellular proliferation than gefitinib. In this *in vitro* metabolic study, we tested the generation of reactive intermediates from SAP using human liver microsomes and a capturing agent (potassium cyanide) to trap the iminium reactive intermediates. The same metabolic reaction was further repeated in the presence of methoxyamine to trap aldehyde intermediates. The identification of SAP metabolites revealed that the hydroxylation metabolic reaction represents the major *in vitro* metabolic pathway occurring at the piperidine moiety. We characterized six *in vitro* phase I metabolites in addition to three reactive intermediates (*i.e.*, two iminiums and one aldehyde), therefore suggesting two probable SAP-bioactivation pathways. We hypothesized that the piperidine ring nitrogen (cyclic tertiary amine) activated the two adjacent α-carbons within the ring. The oxidative dealkylation of the *N*-acetamide group led to an unstable aldehyde that was trapped using methoxyamine, generating an oxime adduct that was detected using liquid chromatography-tandem mass spectrometry (LC-MS/MS). To the best of our knowledge, this is the first study presenting the structural characterization of SAP reactive intermediates.

## Abstract 2

Sapitinib is a competitive ATP inhibitor of EGFR and receptor tyrosine-protein kinase (erbB-2). Two cyano and one oxime adducts, and six *in vitro* metabolites of sapitinib were identified using LC-MS/MS. The bioactivation pathways were characterized.

## Graphical abstract

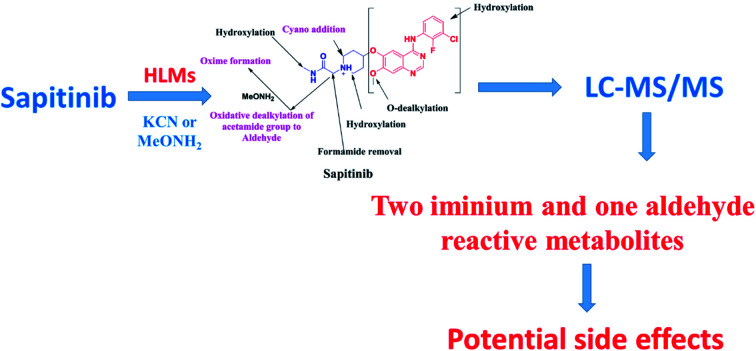

## 1. Introduction

**Fig. 1:**
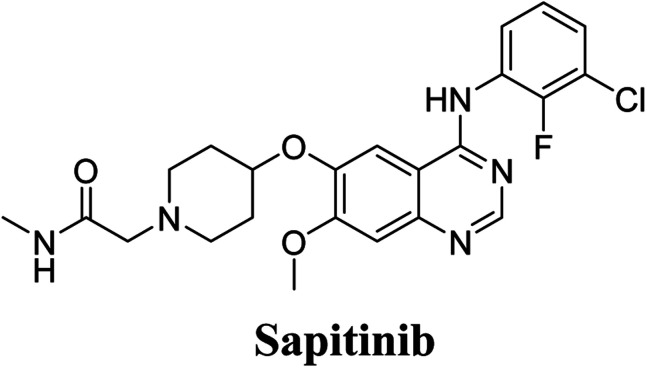
Chemical structure of Sapitinib.

Sapitinib (AZD8931, SAP) is a tyrosine kinase inhibitor (TKI) of the epidermal growth factor receptor (EGFR) family (Fig. 1). SAP is a selective, potent, and competitive ATP inhibitor of EGFR and receptor tyrosine-protein kinase (erbB-2) that met its primary endpoint in phase 2/pre-phase 3 trials.^1^ SAP was found to be a more potent inhibitor of EGF-driven cellular proliferation in various tumor cell lines when compared to gefitinib, the previous first line clinical TKI. SAP uniquely provides similar inhibition of erbB2, erbB3, EGFR, and signaling, and exhibits more antitumor activity in specific preclinical models compared to other narrower spectrum agents with erbB receptor inhibition.^2^

Four adverse effects, *i.e.*, gingival pain, blepharitis, oropharyngeal pain, and catheter-site-related reaction have been already reported by three subjects.^3^ Side effects might be explained by reactive metabolites generated during the metabolism of xenobiotics which can bind to proteins and modify it, a mechanism considered an early step in organ toxicity.^4,5^ Reactive intermediates are usually intermediate compounds formed during the initial stage of metabolism that can lead to various side effects. Because of the unstable nature of reactive species, capturing agents are usually used to generate stable adducts that can then be extracted, separated, and detected by liquid chromatography-tandem mass spectrometry (LC-MS/MS).^6–9^

The systemic name of SAP is 2-[4-({4-[(3-chloro-2-fluorophenyl)amino]-7-methoxy-6-quinazolinyl}oxy)-1-piperidinyl]-*N*-methylacetamide. Its chemical structure contains one piperidine ring linked to an *N*-methyl acetamide functional group (Fig. 1). During phase 1 metabolism, the α-carbon adjacent to the tertiary nitrogen of the piperidine ring is hydroxylated, which leads to the loss of one water molecule, generating unstable and reactive iminium-ion species that can be trapped using cyanide-forming stable adducts. The generation of iminium intermediates has been reported to be the main cause of drug-related toxicity.^9–12^ The *N*-methylacetamide group undergoes metabolism by oxidative dealkylation, generating aldehyde species that are unstable and can be captured using methoxyl amine to form an oxime adduct. Aldehyde intermediates have been considered to be the cause of many side effects.^9,13,14^ These adducts can be characterized, separated, and identified using LC-MS/MS.^6,7,15–18^

The toxic adverse effects associated with SAP are therefore likely caused by the generation of reactive species.^19^ In this context, we investigated the phase I metabolism of SAP in human liver microsomes (HLMs). We identified SAP-generated metabolites and reactive intermediates using LC-MS/MS in product ion scan mode, where compounds were fragmented in the collision cell and fragments detected in the third quadrupole. The qualitative daughter ions (DIs) corresponding to specific fragments of SAP were identified, enabling the characterization of SAP metabolism and prediction of the chemical structures of the formed metabolites and reactive intermediates.

## 2. Material and methods

### 2.1. Materials

All solvents and chemicals were of analytical grade. HPLC-grade water (H_2_O) was generated by a Milli-Q plus filtration system (Millipore, Billerica, MA, USA). SAP was purchased from MedChem Express (NJ, USA). HPLC-grade acetonitrile (ACN), methoxyamine (MeONH_2_), human liver microsomes (HLMs, M0567), ammonium formate (NH_4_COOH), formic acid (HCOOH), and potassium cyanide (KCN) were purchased from Sigma-Aldrich (St. Louis, MO, USA).

### 2.2. Liquid chromatography-tandem mass spectrometry

The chromatographic analysis of SAP HLM-incubation mixtures was carried out using an Agilent 1200 RRLC system (Agilent Technologies). For identification and characterization of SAP metabolites and its reactive intermediates (*i.e.*, cyano and oxime adducts), the LC system was hyphenated to an Agilent 6410 Triple Quadrupole mass spectrometer (Agilent Technologies) equipped with an electrospray ionization (ESI) source. The separation was performed using a C_18_ stationary phase (150 mm × 2.1 mm, 3.5 μm) thermostated at 20 ± 2 °C. The mobile phase was composed of 10 mM ammonium formate at pH 4.2 (solvent A) and ACN (solvent B). The flow rate was 0.25 mL min^−1^. The stepwise-gradient was the following: solvent B (5%; 0–5 min), solvent B (5–40%; 5–40 min), solvent B (40–80%; 40–60 min), and solvent B (80–5%; 60–65 min). A post-run re-equilibration time of 15 min was used. The sample injection volume was 20 μL. The MS experimental parameters were optimized for SAP, its metabolites, and the reactive metabolites using flow injection analysis. Fragmentation of SAP, metabolites, and trapped reactive metabolites were done in the collision cell of the triple quadrupole mass analyzer. Detection was done in ESI positive ion mode.^18,19^ ESI temperature, fragmentor voltage, and capillary tube voltage were set at 350 °C, 135 V, and 4000 V, respectively. The collision energy was fixed at 25 eV for all compounds. Nitrogen (low-purity) was utilized as drying gas at a flow rate of 12 L min^−1^. Nitrogen (high-purity) was used as collision gas at a pressure of 55 psi. Data acquisition and instrument management was carried out using Mass Hunter software (Agilent Technologies).

### 2.3. SAP metabolism in HLMs

SAP (10 μM in DMSO) was incubated with HLMs (1.0 mg mL^−1^) for 2 h in an incubation mixture of Na/K phosphate buffer (50 mM, pH 7.4) and magnesium chloride (3.3 mM) in a shaking water bath (37 °C). The metabolism was initiated by adding a solution of 1.0 mM NADPH and quenched with addition of 2 mL ice-cold ACN leading to protein precipitation. After centrifugation at 9000×*g* for 10 min at 4 °C, the supernatant was collected and evaporated under a gentle stream of nitrogen. The residue was then reconstituted in the mobile phase and transferred into HPLC vials prior to LC-MS/MS analysis.^20–23^

### 2.4. Generation of reactive intermediates

SAP was incubated in HLMs using KCN (1.0 mM) or MeONH_2_ (2.5 mM) as trapping agents for iminium species or aldehyde intermediates, respectively. The trapping agents were added before the initiation of metabolism (NADPH addition). Experiments were repeated three independent times for method validation.

### 2.5. Identification of SAP reactive metabolites

Characterization of SAP and its related metabolites in the incubation mixture was done using extracted ion chromatograms of the daughter ions' (DIs) chromatographic peaks. Among those, DIs representing a substructure of the parent metabolite were considered. These fragments enabled the prediction of the compound sites exposed to phase I metabolism as well as the chemical structures of the formed SAP metabolites.

## 3. Results and discussion

### 3.1. SAP fragmentation pattern

**Scheme 1:**
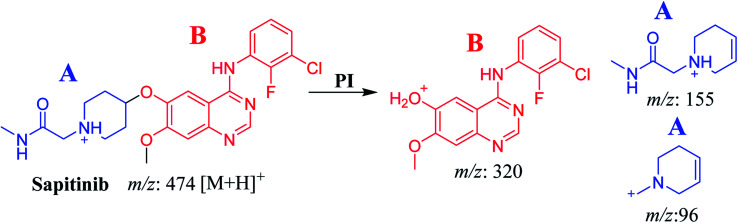
Fragments (DIs) observed after fragmentation of SAP.

**Fig. 2:**
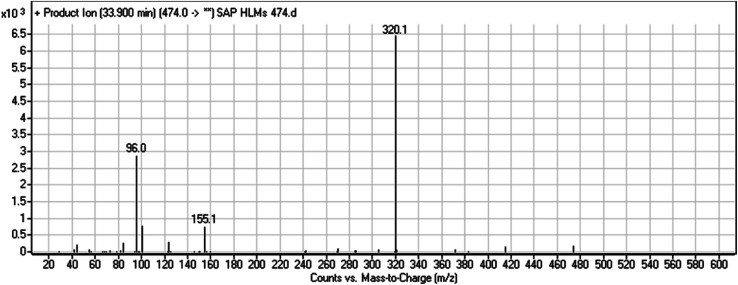
SAP PI mass spectrum. SAP, sapitinib; PI, product ion.

The chemical structure of SAP is shown as two substructures, *i.e.*, A (blue) and B (red) (Scheme 1), to facilitate the identification of metabolic pathways characterized by changes in relevant DIs. SAP chromatographic peak was detected at 33.9 min. Fragmentation of the SAP ion detected at *m*/*z* 474 (corresponding to the [M + H]^+^ form) resulted in three qualitative DIs detected at *m*/*z* 320, *m*/*z* 155, and *m*/*z* 96 (Fig. 2). These DIs showed a relevant substructure that enabled the determination of phase I metabolism pathways in HLMs and prediction of related SAP metabolites structures (Scheme 1).

### 3.2. Identification of SAP-related metabolites

We identified hydroxylation reaction as being the major pathway in *in vitro* SAP metabolism. Indeed, two cyano and one oxime adducts were identified after metabolic incubation with HLMs in the presence of 1.0 mM KCN or 2.5 mM MeONH_2_, respectively (Table 1).

**Table 1:** *In vitro* reactive SAP metabolites

	MS precursor ion (*m*/*z*)	Most abundant DIs (*m*/*z*)	RT^a^ (min)	Metabolic pathways	
				Substructure A	Substructure B
SAP	474	320, 155, 96	33.9		
M1	460	306, 155, 96	32.7	No	*O*-demethylation
M2	461	403, 320, 142	32.6	Oxidation of *N*-acetamide to *N*-acetic acid	No
M3	403	320, 84	32.9	*N*-dealkylation of acetamide group	No
M4	490	336, 155, 96	29.3	No	Hydroxylation
M5	490	460, 415, 320, 171, 96	32.7	Hydroxylation at methyl group of *N*-acetamide group	No
M6	490	415, 372, 320, 171, 96	35.1	Hydroxylation at piperidine group	No
M7	499	472, 320, 153, 94	46.3	Cyano addition at piperidine ring	No
M8	442	415, 320, 96	46.6	Cyano addition with formamide removal	No
M9	460	320, 141, 87	32.9	Oxime adduct formation with oxidative dealkylation of acetamide group	No

a RT, retention time; DI, daughter ion.

#### 3.2.1. HLMs metabolite detected at *m*/*z* 460 (M1)

**Fig. 3:**
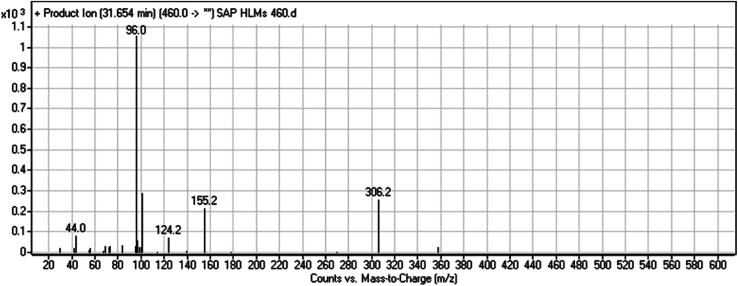
M1 PI mass spectrum.

**Scheme 2:**
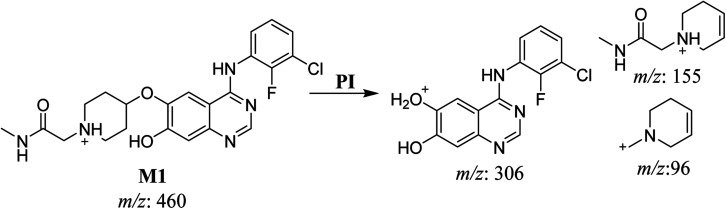
Fragments (DIs) observed after fragmentation of M1.

The SAP metabolite detected at *m*/*z* 460 (M1) eluted at 31.7 min. The fragmentation of M1 ion at *m*/*z* 460 (corresponding to the [M + H]^+^ form) resulted in three DIs detected at *m*/*z* 306, *m*/*z* 155, and *m*/*z* 96 (Fig. 3). Compared with the fragmentation of SAP, the two DIs detected at *m*/*z* 155 and *m*/*z* 96 did not show any relevant metabolic pathway in the A substructure, whereas the DI detected at *m*/*z* 306 exhibited a loss of 14 Da, indicating an *O*-demethylation which is the only possible metabolic reaction in the B substructure (Scheme 2).

#### 3.2.2. HLMs metabolite detected at *m*/*z* 461 (M2)

**Fig. 4:**
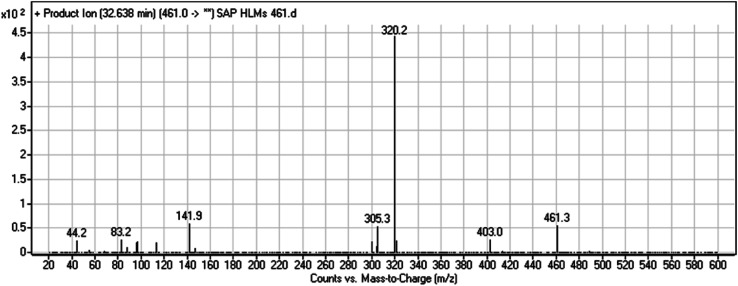
M2 PI mass spectrum.

**Scheme 3:**
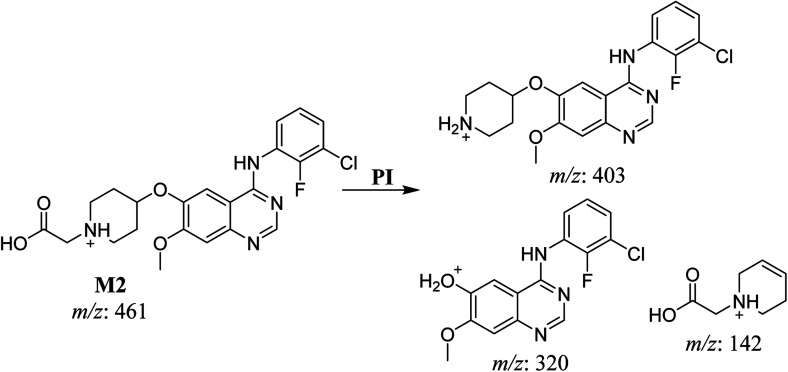
Fragments (DIs) observed after fragmentation of M2.

The SAP metabolite detected at *m*/*z* 461 (M2) eluted at 32.6 min. The fragmentation of M2 ion at *m*/*z* 461 (corresponding to the [M + H]^+^ form) resulted in three DIs detected at *m*/*z* 403, *m*/*z* 320, and *m*/*z* 142 (Fig. 4). Compared with SAP fragmentation, the DI detected at *m*/*z* 320 did not any relevant metabolic pathway in the B substructure, whereas the DI detected at *m*/*z* 141 indicated an oxidation of the *N*-acetamide group to form *N*-acetic acid, matching with the structure of the DI detected at *m*/*z* 403 (Scheme 3).

#### 3.2.3. HLMs metabolite detected at *m*/*z* 403 (M3)

**Fig. 5:**
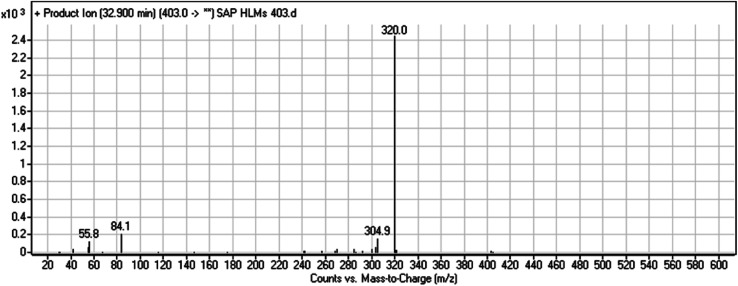
M3 PI mass spectrum. SAP, sapitinib; PI, product ion.

**Scheme 4:**
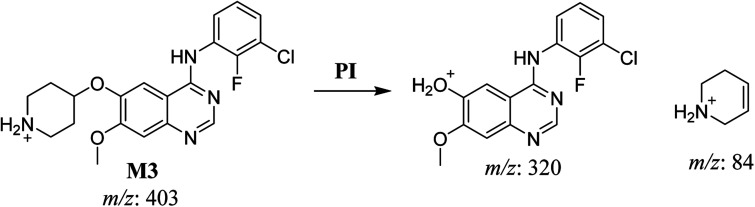
Fragments (DIs) observed after fragmentation of M3.

The SAP metabolite detected at *m*/*z* 403 (M3) eluted at 32.9 min. The fragmentation of M3 ion at *m*/*z* 403 (corresponding to the [M + H]^+^ form) resulted in two qualitative DIs detected at *m*/*z* 320 and *m*/*z* 84 (Fig. 5). Compared with SAP fragmentation, the DI detected at *m*/*z* 320 did not show any relevant metabolic pathway in the B substructure, whereas the DI detected at *m*/*z* 84 revealed *N*-dealkylation of the acetamide group (Scheme 4).

#### 3.2.4. HLMs metabolites detected at *m*/*z* 490 (M4, M5, and M6)

**Fig. 6:**
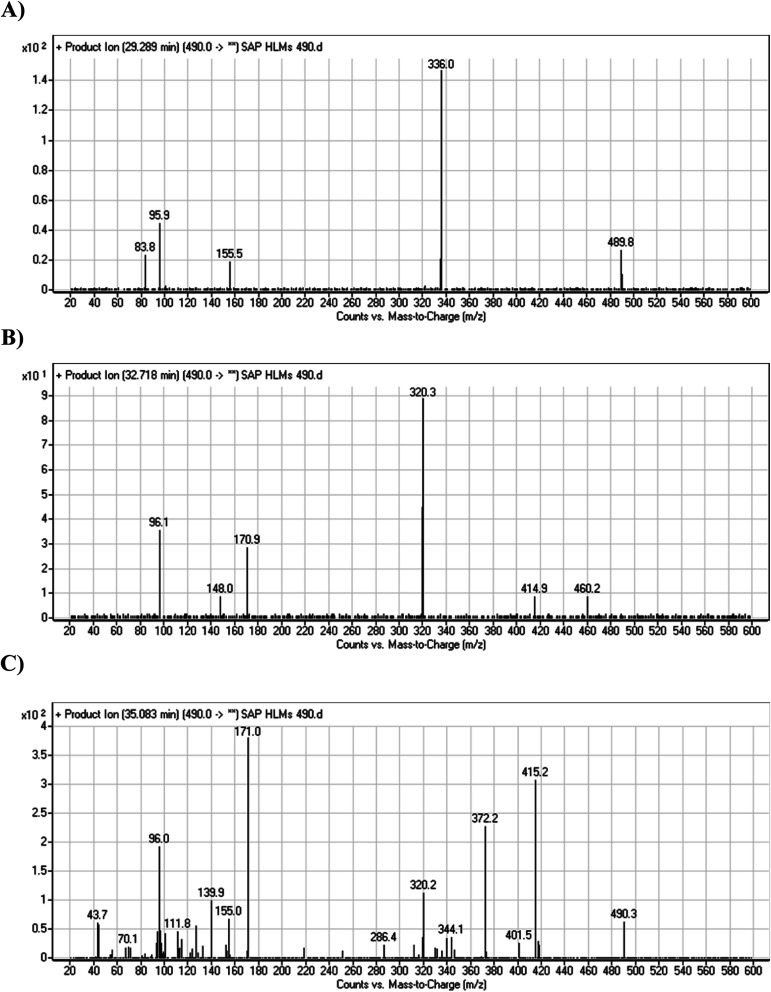
PI mass spectrum of M4 (A), M5 (B), and M6 (C).

Many oxidized SAP metabolites were detected at *m*/*z* 490 (corresponding to the [M + H]^+^ form) with different elution times, with major peaks detected at 29.3 min, 32.7 min, and 35.1 min. The fragmentation of the ions detected at *m*/*z* 490 resulted in various DIs (Fig. 6A–C), suggesting that hydroxylation happened at multiple sites on the SAP molecule.

**Scheme 5:**
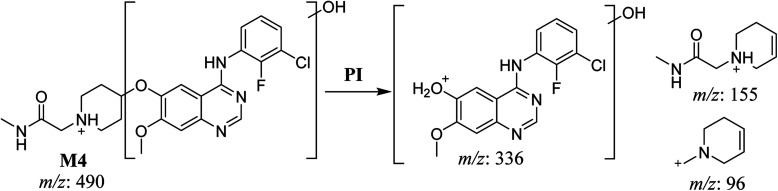
Fragments (DIs) observed after fragmentation of M4.

The fragmentation of the first ion detected at *m*/*z* 490 (corresponding to the [M + H]^+^ form), with a retention time of 29.3 min (M4), resulted in three DIs detected at *m*/*z* 336, *m*/*z* 155, and *m*/*z* 96 (Fig. 6A). Compared with SAP fragmentation, the DIs detected at *m*/*z* 155 and *m*/*z* 96 did not show relevant metabolic pathway in the A substructure, whereas the DI detected at *m*/*z* 336 exhibited a loss of 16 Da that pointed out a hydroxylation in the B substructure (Scheme 5).

**Scheme 6:**
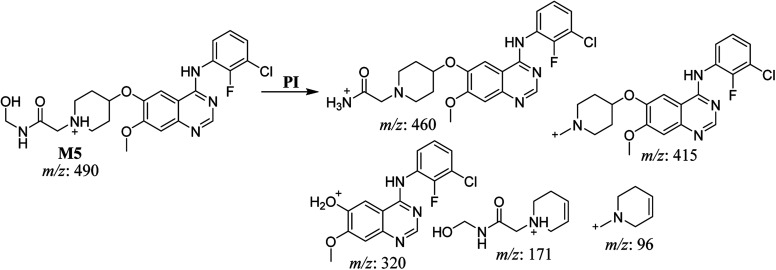
Fragments (DIs) observed after fragmentation of M5.

The fragmentation of the second ion detected at *m*/*z* 490 (corresponding to the [M + H]^+^ form), with a retention time of 32.7 min (M5) resulted in five DIs detected at *m*/*z* 460, *m*/*z* 415, *m*/*z* 320, *m*/*z* 171, and *m*/*z* 96 (Fig. 6B). Compared with SAP fragmentation, the DIs detected at *m*/*z* 320 did not show any relevant metabolic pathway in the B substructure, whereas the DI detected at *m*/*z* 171 exhibited an addition of 16 Da that revealed an hydroxylation in the A substructure, which matched with the structure of the DI detected at *m*/*z* 415. The DI detected at *m*/*z* 460 showed a hydroxyl methyl loss that revealed a hydroxylation in the methyl group (Scheme 6).

**Scheme 7:**
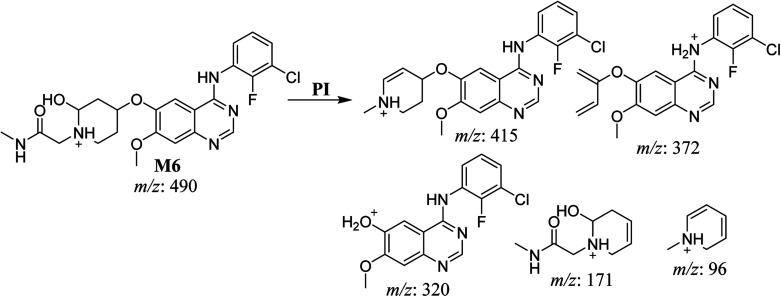
Fragments (DIs) observed after fragmentation of M6.

The fragmentation of the third ion detected at *m*/*z* 490 (corresponding to the [M + H]^+^ form), with a retention time of 35.1 min (M6), resulted in five DIs detected at *m*/*z* 415, *m*/*z* 372, *m*/*z* 320, *m*/*z* 171, and *m*/*z* 96 (Fig. 6C). Compared with the SAP fragmentation, the DIs detected at *m*/*z* 320 did not show any relevant metabolic pathway in the B substructure, whereas the DI detected at *m*/*z* 171 exhibited an addition of 16 Da that revealed an hydroxylation in the A substructure, which matched with the structure of the DI detected at *m*/*z* at 415. The DI detected at *m*/*z* 372 showed a Retro-Diels–Alder reaction, indicating a hydroxylation of the piperidine group, which matched with the structure of the DI detected at *m*/*z* at 96 (Scheme 7).

#### 3.2.5. HLMs reactive metabolite detected at *m*/*z* 499 (M7)

The metabolic bioactivation of SAP created an electron-deficient center on the molecule, which was attacked by a cyano-nucleophile (electron-rich), generating reactive iminium species. The presence of DI showing a neutral loss of 27 Da, corresponding to a hydrogen cyanide unit, was used to detect potential cyano adducts as this fragment is characteristic for cyano adducts.^10,12^ During bioactivation of similar cyclic tertiary amine-containing drugs,^24,25^ the piperidine-ring carbons are supposed to be the bioactive center.

**Fig. 7:**
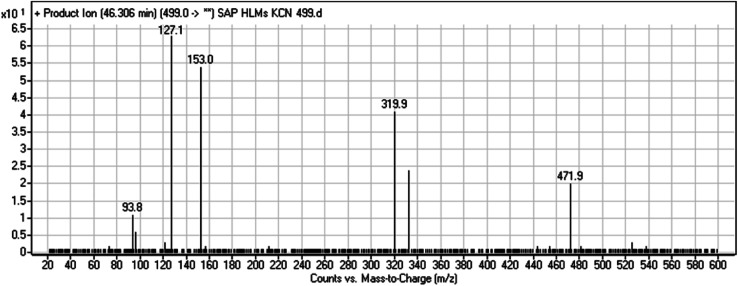
M7 PI mass spectrum.

**Scheme 8:**
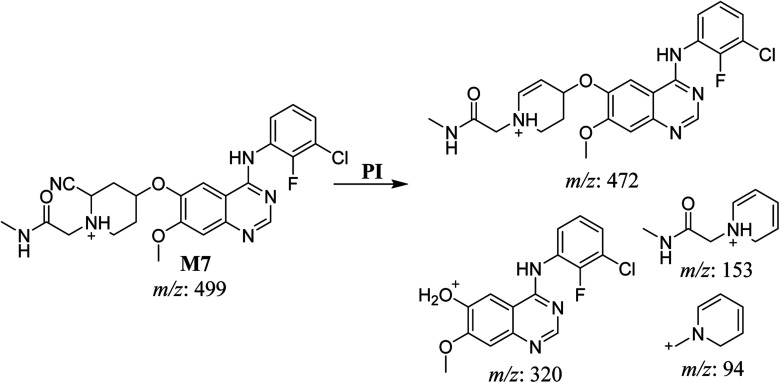
Fragments (DIs) observed after fragmentation of M7.

A cyano adduct of SAP metabolite detected at *m*/*z* 499 (corresponding to the [M + H]^+^ form) eluted at 46.3 min (M7). The fragmentation of M7 ion resulted in four DIs detected at *m*/*z* 472, *m*/*z* 320, *m*/*z* 153, and *m*/*z* 94 (Fig. 7). The DI detected at *m*/*z* 472 showed a neutral loss of a hydrogen cyanide unit that confirmed the addition of a cyano moiety. Compared with SAP fragmentation, the DIs detected at *m*/*z* 320 did not show any relevant metabolic pathway in the B substructure, whereas the DI detected at *m*/*z* 153 showed and HCN loss, indicating cyano addition at the piperidine group in the A substructure, matching with the structure of the DI detected at *m*/*z* 94 (Scheme 8).

#### 3.2.6. HLMs reactive metabolite detected at *m*/*z* 442 (M8)

**Fig. 8:**
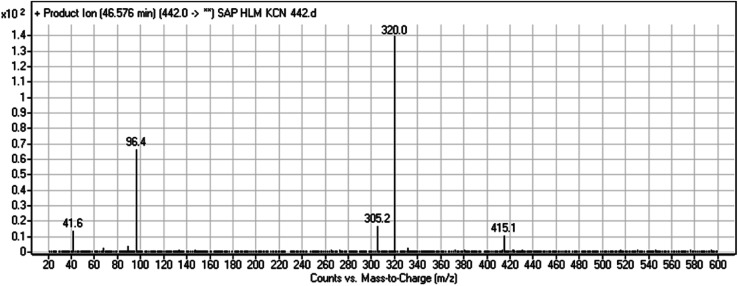
M8 PI mass spectrum.

**Scheme 9:**
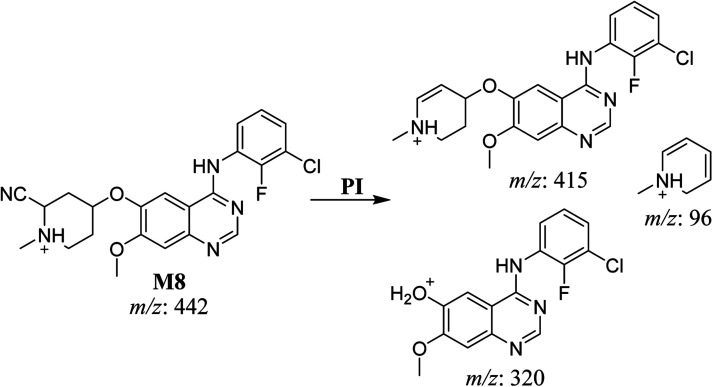
Fragments (DIs) observed after fragmentation of M8.

The cyano adduct of SAP metabolite detected at *m*/*z* 442 (corresponding to the [M + H]^+^ form) eluted at 46.5 min (M8). The fragmentation of M8 ion resulted in three DIs detected at *m*/*z* 415, *m*/*z* 320, and *m*/*z* 96 (Fig. 8). The DI detected at *m*/*z* 415 showed a neutral loss of a hydrogen cyanide unit that confirmed the addition of a cyano moiety. Compared with SAP fragmentation, the DI detected at *m*/*z* 320 did not show and relevant metabolic pathway in the B substructure, whereas the DI detected at *m*/*z* 415 showed a loss of 27 Da, indicating the loss of an HCN unit characteristic of cyano addition at the piperidine group in the A substructure, matching with the structure of the DI detected at *m*/*z* 96 (Scheme 9).

#### 3.2.7. HLMs reactive metabolite detected at *m*/*z* 460 (M9)

**Fig. 9:**
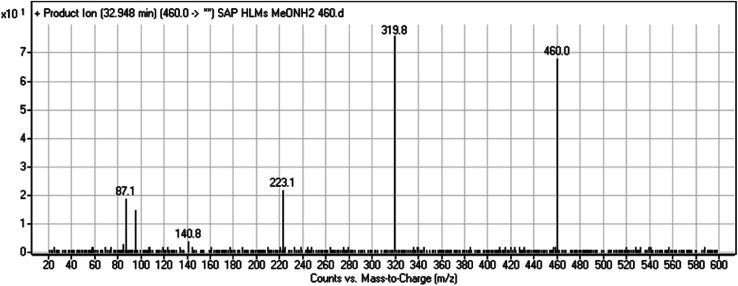
M9 PI mass spectrum.

**Scheme 10:**
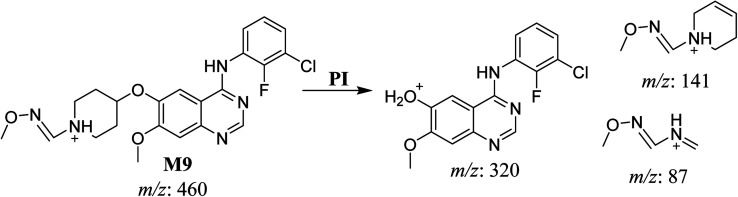
Fragments (DIs) observed after fragmentation of M9.

The SAP metabolite detected at *m*/*z* 460 eluted at 32.9 min (M9). The fragmentation of M9 ion resulted in three DIs detected at *m*/*z* 320, *m*/*z* 141, and *m*/*z* 87 (Fig. 9). Compared with SAP fragmentation, the DI detected at *m*/*z* 320 did not show any relevant metabolic pathway in the B substructure. On the other hand, the DI detected at *m*/*z* 141 revealed oxidative dealkylation of an acetamide group, leading to the generation in the A substructure of an aldehyde intermediate that formed an oxime adduct with MeONH_2_, matching with the structure of the DI detected at *m*/*z* 87 (Scheme 10).

### 3.3. Bioactivation pathways of SAP

**Scheme 11:**
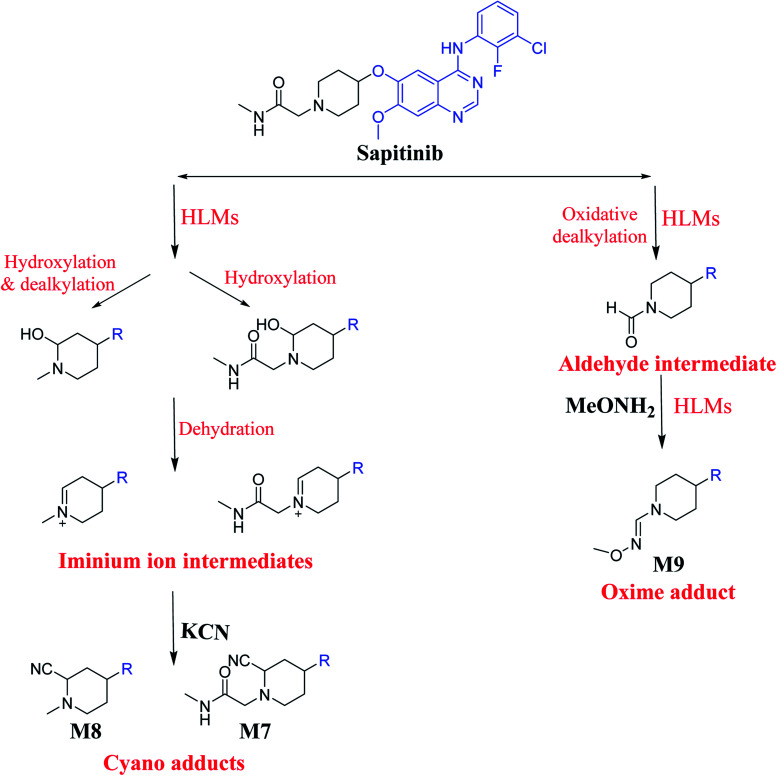
Suggested metabolism for the generation of iminium and aldehyde intermediates of SAP.

The presence of M7 and M8 cyano adducts underlined the bioactivation of the piperidine ring (cyclic tertiary amine) during SAP metabolism. Indeed, the hydroxylation metabolic reaction at α-carbons linked to the tertiary nitrogen of piperidine ring followed by water loss led to the formation of reactive iminium intermediates that were trapped with cyanide to form the stable cyano adducts M7 and M8. The detection of the M9 oxime confirmed the formation of an aldehyde intermediate. Specifically, the oxidative dealkylation of the acetamide group generated an unstable aldehyde intermediate that was trapped by MeONH_2_, forming the stable oxime M9. Two cyano and one oxime adducts were characterized using LC-MS/MS. Scheme 11 shows the pathways involved in SAP bioactivation during its metabolism.

## 4. Conclusion

**Fig. 10:**
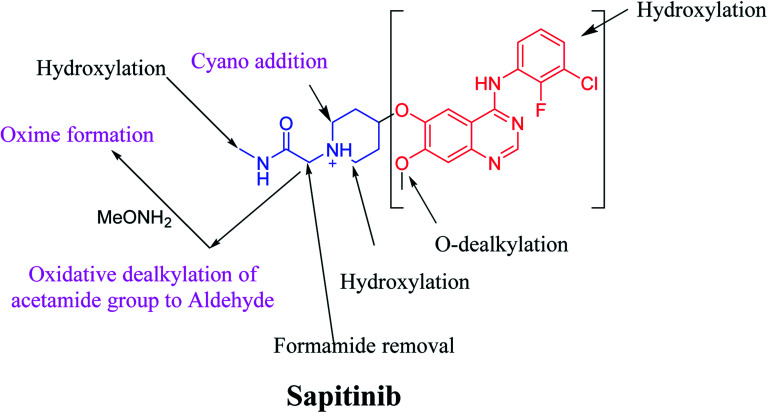
Metabolic and bioactivation pathways of SAP.

We characterized six SAP metabolites using LC-MS/MS in addition to two iminium ions and one aldehyde reactive metabolite. We were able to define two bioactivation pathways for SAP (Fig. 10) from these results (Scheme 11). First, the piperidine-ring carbons were bioactivated during SAP metabolism and captured by cyanide ions. Second, the oxidative dealkylation of the acetamide group led to the formation of an aldehyde that was attacked by MeONH_2_, generating an oxime adduct. The generation of these reactive intermediates in SAP metabolism might be associated with its reported side effects. This study therefore provides relevant insights for the development of second-generation drugs with improved safety profiles. Building new drugs by blocking SAP-bioactivation center or characterization of isosteric replacements for the attacked hydrogen atoms could indeed lead to a decrease in side effects while keeping SAP activity intact.

## Conflicts of interest

None.

## Abbreviations

ACN: Acetonitrile
DI: Daughter ion
EGFR: Epidermal growth factor receptor
ESI: Electrospray ionization
FI: Fragment ion
HLMs: Human liver microsomes
LC-MS/MS: Liquid chromatography-tandem mass spectrometry
KCN: Potassium cyanide
MeONH_2_: Methoxyamine
PI: Product ion
SAP: Sapitinib
TKI: Tyrosine kinase inhibitor

## Supplementary Material

RA-009-C9RA03926K-s001
