# Supplementary material for: Sapitinib: reactive intermediates and bioactivation pathways characterized by LC-MS/MS
Source: RSC Adv. 2019 Oct 16;9(57):32995–3006. doi: 10.1039/c9ra03926k (PMC9073192; doi:10.1039/c9ra03926k)
Supplement: RA-009-C9RA03926K-s001 [file RA-009-C9RA03926K-s001.pdf]

Electronic Supplementary Material (ESI) for RSC Advances.
This journal is © The Royal Society of Chemistry 2019

Supplementary data

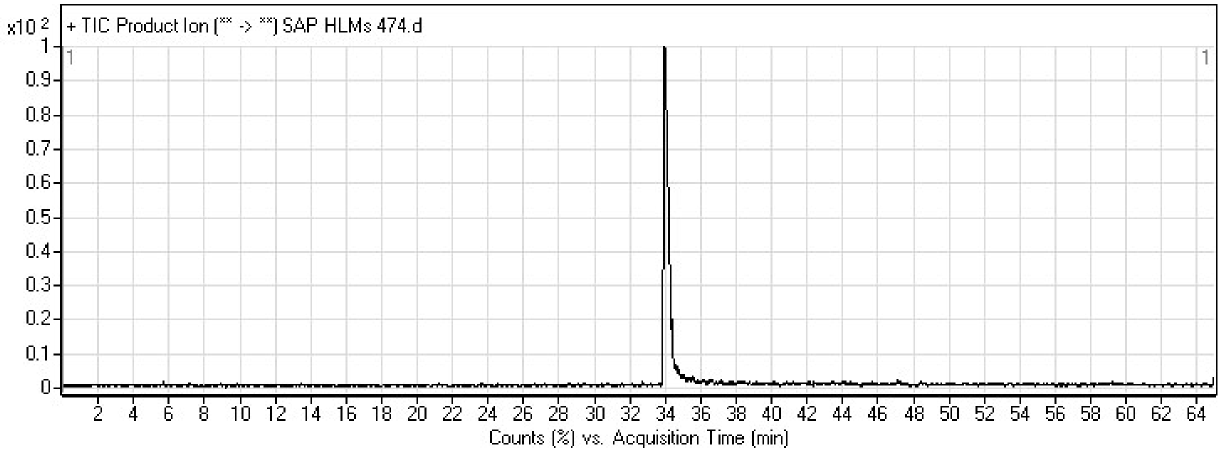

Fig. S1. SAP PI chromatogram.
SAP, sapitinib; PI, product ion.

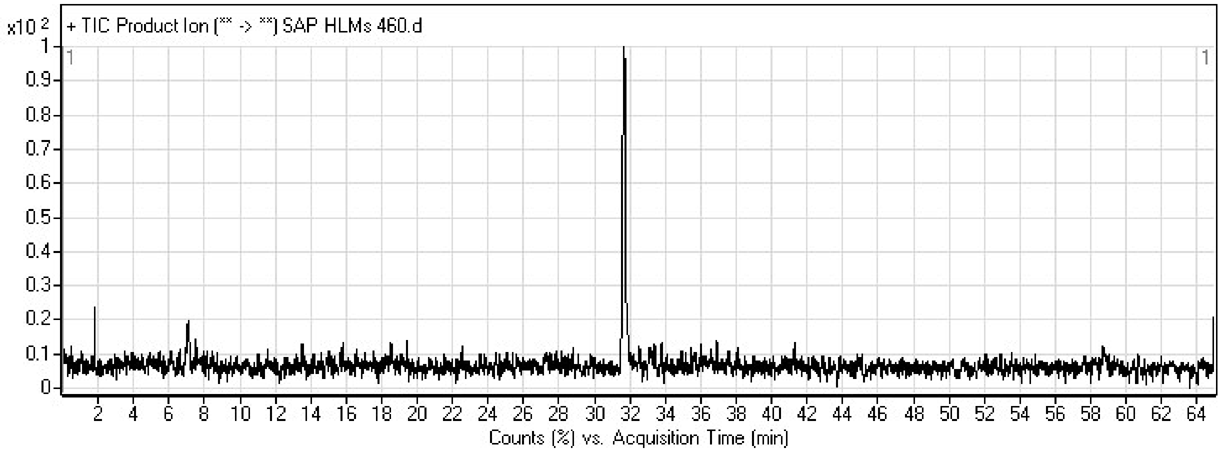

Fig. S2. M1 PI chromatogram.

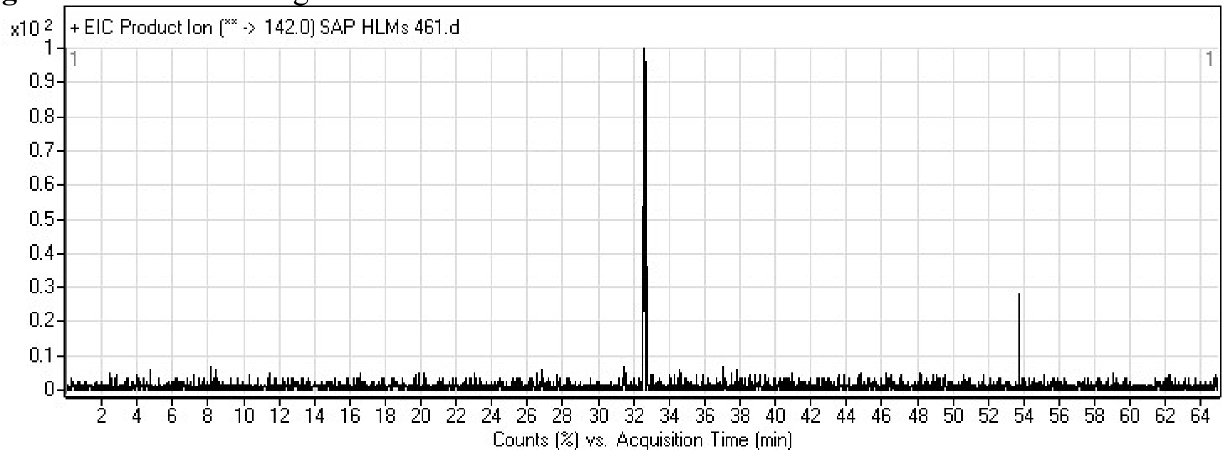

Fig. S3. M2 PI chromatogram.

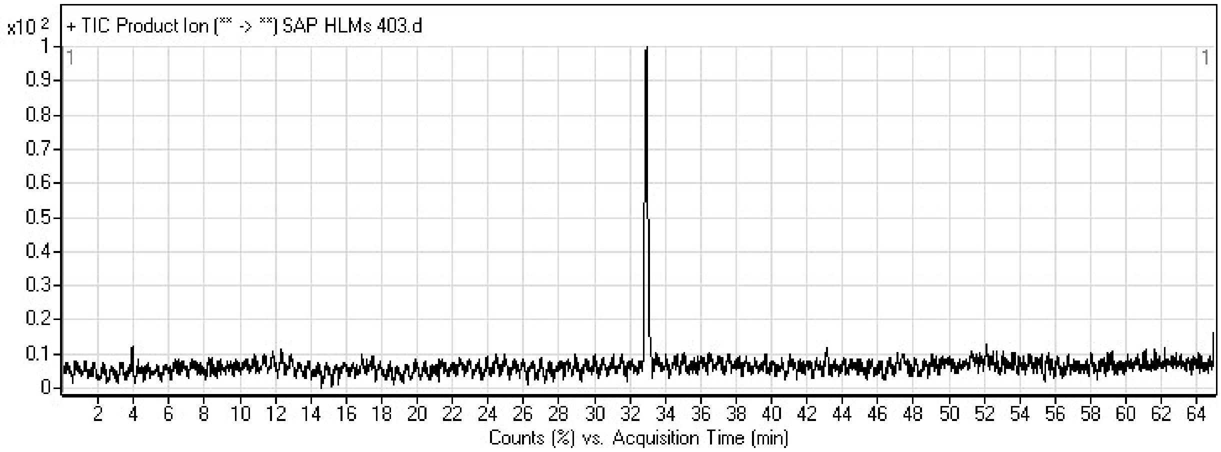

Fig. S4. M3 PI chromatogram.

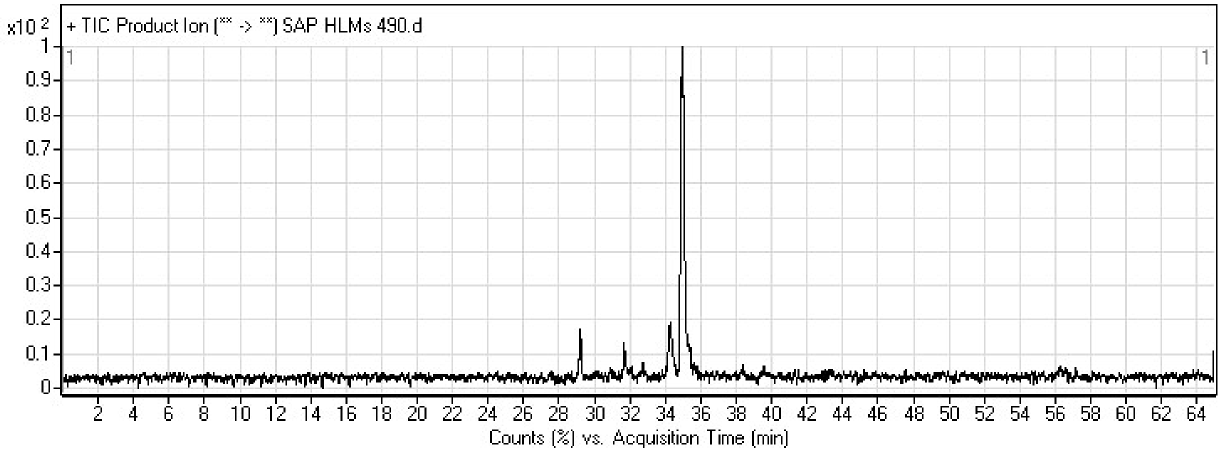

Fig. S5. M4, M5 and M6 PI chromatogram.

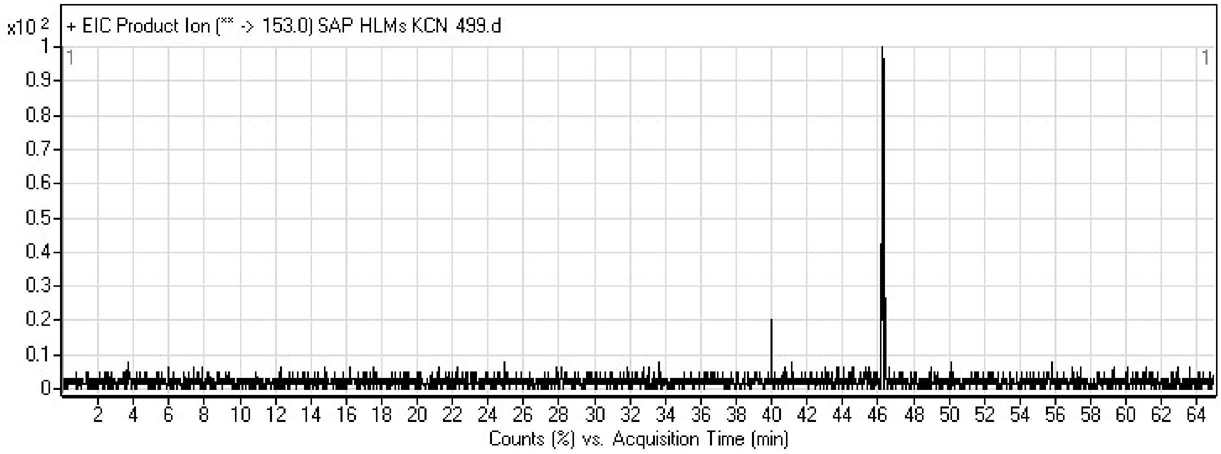

Fig. S6. M7 PI chromatogram.

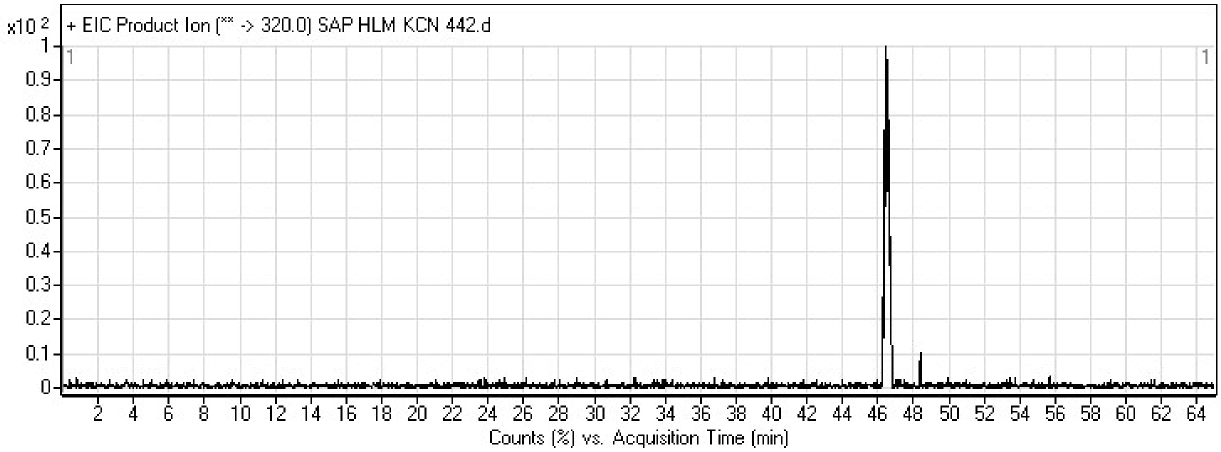

Fig. S7. M8 PI chromatogram.

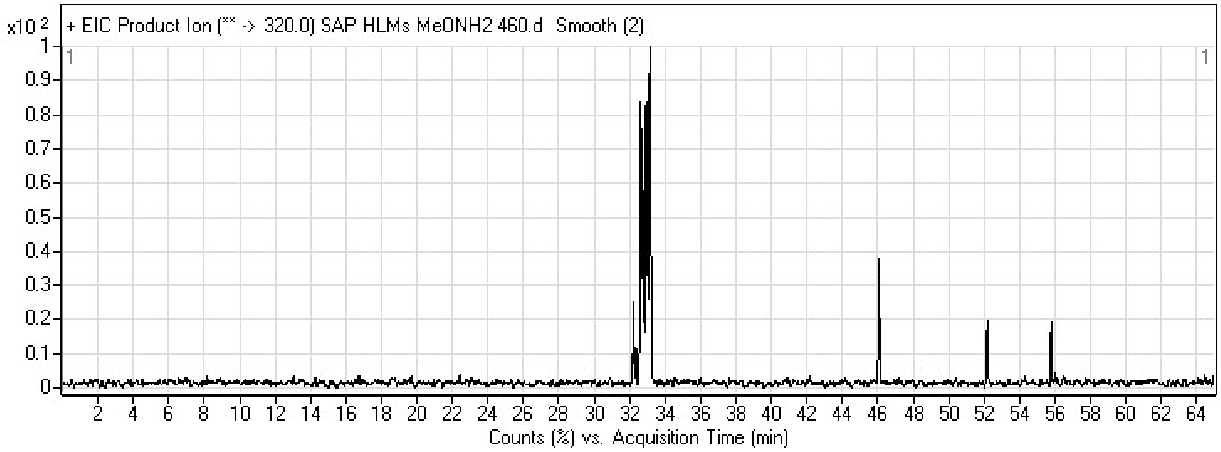

Fig. S8. M9 PI chromatogram.
